# The optimal extent of lymph node dissection for adenocarcinoma of the esophagogastric junction differs between Siewert type II and Siewert type III patients

**DOI:** 10.1007/s10120-014-0364-0

**Published:** 2014-03-22

**Authors:** Hironobu Goto, Masanori Tokunaga, Yuichiro Miki, Rie Makuuchi, Norihiko Sugisawa, Yutaka Tanizawa, Etsuro Bando, Taiichi Kawamura, Masahiro Niihara, Yasuhiro Tsubosa, Masanori Terashima

**Affiliations:** 1 Division of Gastric Surgery, Shizuoka Cancer Center, 1007 Shimonagakubo, Nagaizumi-cho, Sunto-gun, Shizuoka, 411-8777 Japan; 2 Division of Esophageal Surgery, Shizuoka Cancer Center, Shizuoka, Japan

**Keywords:** Gastric cancer, Adenocarcinoma of the esophagogastric junction, Siewert type II, Siewert type III

## Abstract

**Background:**

The incidence of adenocarcinoma of the esophagogastric junction (AEG) has been increasing worldwide. We investigated the clinicopathological characteristics of patients with Siewert type II and III AEGs and clarified the optimal intra-abdominal lymph node dissection in these patients.

**Methods:**

This study included 132 patients with AEG who underwent curative resection at Shizuoka Cancer Center from September 2002 to December 2012. We used the index of estimated benefit from lymph node dissection (IEBLD) to assess the efficacy of lymph node dissection of each station. The clinicopathological characteristics and IEBLDs of each station were compared between patients with Siewert type II and III AEGs.

**Results:**

We analyzed 92 patients with Siewert type II AEG and 40 patients with Siewert type III AEG. The incidence of lymph node metastasis was high in both groups (64.1 % in type II AEG and 75.0 % in type III AEG). The 5-year survival rates were similar for the patients with Siewert type II and III AEGs, at 54.0 and 53.4 %, respectively. The IEBLDs of stations located near the esophagogastric junction were generally high in both groups, while the IEBLDs of lower perigastric lymph nodes were higher in Siewert type III than in Siewert type II AEG cases.

**Conclusions:**

The IEBLDs were similar between Siewert type II and III AEGs at all stations except for lower perigastric lymph nodes. Total gastrectomy should be selected as a standard treatment for Siewert type III AEG, whereas in Siewert type II AEG, preservation of the distal part of the stomach may be an acceptable procedure.

## Introduction

The incidence of adenocarcinoma of the esophagogastric junction (AEG) has been increasing recently in both Eastern and Western countries [1]. In Eastern countries, westernized lifestyle habits and the increased incidence of gastroesophageal reflux disease are thought to be possible reasons, with the incidence of AEG likely to increase further [2].

Siewert et al. [3] classified AEG into three subgroups according to the location of the tumor’s epicenter. Siewert type I AEG is the most prevalent type in Western countries and is generally treated as an esophageal cancer [4]. The standard surgical procedure for Siewert type I AEG is a subtotal esophagectomy with proximal gastrectomy through thoracotomy [5]. Siewert type II and type III AEGs are more common than Siewert type I AEG in Eastern countries and are mostly treated as a gastric cancer with a trans-hiatal approach [6].

In contrast, the seventh edition of TNM classification categorized AEG as an esophageal cancer irrespective of the Siewert type, and indeed, a current concern of surgeons, particularly those in East Asia, is whether Siewert type II and III AEGs should be regarded and thus surgically approached as the same tumor [7].

Recently, the value of intra-abdominal and mediastinal lymph node dissection for AEG has been investigated [6, 8–10]. We also investigated clinicopathological characteristics of Siewert type II AEG to clarify the optimal intra-abdominal lymph node dissection and reported that splenic hilar lymph node dissection might be omitted [11]. However, most reports, including ours, focused on Siewert type II AEG, and few have investigated the value of lymph node dissection for Siewert type III AEG [12, 13]. Accordingly, the optimal extent of lymph node dissection for Siewert type III AEG remains unclear. Therefore, this study aimed to clarify any required differences in optimal intra-abdominal lymph node dissection between Siewert type II and III AEGs.

## Materials and methods

### Patients

From September 2002 to December 2012, 3,185 patients with gastric cancer underwent gastrectomy at Shizuoka Cancer Center. Of these, 176 patients underwent gastrectomy with lymph node dissection for Siewert type II or III AEG. Patients who received preoperative chemotherapy (10 patients) and those who underwent non-curative gastrectomy (R1 or R2, 38 patients) were excluded, and the remaining 132 patients were included in the present study.

The International Union Against Cancer (UICC) TNM staging system for esophageal cancer was used for tumor staging [7], while the lymph node stations were numbered as defined by the Japanese Gastric Cancer Association (JGCA) [14]. Tumor histology was also evaluated according to the JGCA classification [14], with well and moderately differentiated tubular adenocarcinoma and papillary adenocarcinoma classified as differentiated-type carcinomas, and poorly differentiated adenocarcinoma, signet ring cell carcinoma, and mucinous carcinoma classified as undifferentiated-type carcinomas.

We collected patient characteristics as well as pathological and surgical findings from our database records and individual patient electronic medical records if necessary. The data collection and analysis were approved by the institutional review board.

### Treatment of resected specimens

Immediately after the surgery, we photographed the resected specimen. In this study, a surgeon (H.G.) reviewed these photos retrospectively and classified every patient as Siewert type II or type III. The surgeons also assigned the lymph node stations postoperatively from the en bloc specimen. The standard technique for histological assessment of lymph nodes was hematoxylin and eosin staining of sections from the maximal cut surface.

### Evaluation of the therapeutic value of intra-abdominal lymph node dissection

We adopted the index of estimated benefit from lymph node dissection (IEBLD), a concept proposed by Sasako et al. [15], to assess the efficacy of lymph node dissection of each station. This index is calculated by multiplying the frequency of lymph node metastasis to each station by the 5-year survival rate of patients with positive lymph nodes at each station. The incidence of metastasis and the 5-year survival rate of patients with positive nodes were calculated independently for each lymph node, without any reference to the overall pathological nodal stage.

### Statistics

All statistical analysis was carried out using R statistics version 2.13.1. All continuous variables are presented as the median (range). Statistical analyses were performed using Fisher’s exact test and the Mann-Whitney test. The Kaplan-Meier method was used to estimate survival curves. A *P* value <0.05 was considered significant.

## Results

### Patient characteristics

Table 1 lists the clinicopathological characteristics of the patients, comprising 92 with Siewert type II AEG and 40 with Siewert type III AEG. There were no significant differences in age, sex, histological type, circumferential distribution, or surgical approach between Siewert type II and III AEGs. Type 3 tumors were the most common macroscopic type in Siewert type III AEGs (22 patients, 55.0 %). In addition, patients with Siewert type III AEG showed more advanced disease, larger tumor diameters and depth, and a more advanced pathological stage than those with Siewert type II AEG, although the incidence of lymph node metastasis was high in both groups (64.1 % in type II AEG and 75.0 % in type III AEG). When we stratified patients according to the tumor depth, the incidence of lymph node metastasis was 42.9 % (42.3 % in Siewert type II and 50.0 % in Siewert type III) in patients with pT1 disease and 74.0 % (72.7 % in Siewert type II and 76.3 % in Siewert type III) in patients with pT2–4 disease. In addition, tumor diameter was larger in Siewert type II AEG patients with lymph node involvement compared to those without (55.0 vs. 38.0 mm, *P* = 0.019), whereas there was no such association between tumor diameter and nodal status among Siewert type III AEG patients (69.0 vs. 63.5 mm, *P* = 0.827).

**Table 1:** Characteristics of 121 patients with Siewert type II and type III adenocarcinoma of the esophagogastric junction

Parameters	Siewert type II (*n* = 92)	Siewert type III (*n* = 40)	*P* value
Age			
Median (range), years	68.0 (27–86)	68.0 (28–82)	0.666
Sex			
Male	72	33	0.646
Female	20	7	
Tumor size			
Median (range), mm	43.5 (0–145)	67.5 (0–165)	<0.001
Length of esophageal involvement			
Median (range), mm	10.0 (1–45)	10.0 (2–27)	0.233
Macroscopic type			
Type 0	34	3	0.001
Type 1	12	7	
Type 2	18	8	
Type 3	26	22	
Type 4	2	0	
Circumferential distribution			
Anterior wall	6	5	0.179
Posterior wall	13	4	
Greater curvature	8	5	
Lesser curvature	47	24	
Circular	18	2	
Histological type			
Differentiated	61	27	0.893
Undifferentiated	31	13	
Type of surgery			
Total gastrectomy	73	38	0.036
Proximal gastrectomy	19	2	
Approach			
Abdominal	82	37	0.754
Thoracoabdominal	10	3	
Tumor depth (pathological)			
T1	26	2	0.002
T2	15	2	
T3	34	24	
T4	17	12	
Nodal status (pathological)			
N0	33	10	0.094
N1	23	5	
N2	18	12	
N3	18	13	
Stage (pathological)			
I	33	3	0.003
II	26	11	
III	29	24	
IV	4	2	

### Survival outcomes

**Fig. 1:**
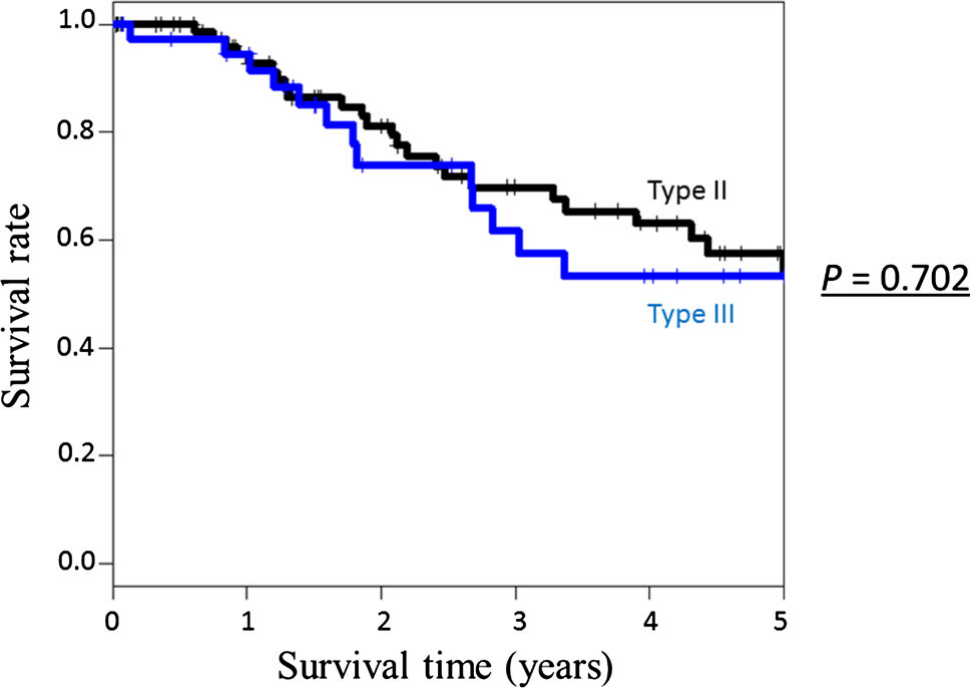
Overall survival in patients with Siewert type II and III AEG. The 5-year survival rate was 54.0 % for Siewert type II AEG and 53.4 % for Siewert type III AEG. The difference was not significant (*P* = 0.702)

Figure 1 shows the survival curves of all patients. The 5-year survival rate was 54.0 % for patients with Siewert type II AEG and 53.4 % for those with Siewert type III AEG (*P* = 0.702). The median follow-up periods of patients and survivors were 23.7 and 21.5 months, respectively, for Siewert type II AEG and 22.3 and 30.8 months, respectively, for Siewert type III AEG. Table 2 shows the first recurrence site. The first recurrence site was not different between Siewert type II and III AEGs: lymph node recurrence was the most frequently observed, followed by peritoneal recurrence, liver metastasis, and local recurrence.

**Table 2:** The first recurrence site in patients with Siewert type II and III adenocarcinoma of the esophagogastric junction

	Type 2	Type 3
Lymph node	16	9
Peritoneum	8	3
Liver	5	2
Lung	2	1
Anastomosis site	1	1
Pleura	1	0
Brain	0	1

The frequency of metastasis of each regional lymph node, the 5-year survival rate of the patients with nodal involvement, and the IEBLD for each station are shown in Tables 3 and 4. The IEBLDs of stations located near the EGJ were generally high. The index was higher than 5 in stations 1 (right paracardia), 3 (lesser curvature), and 7 (along the left gastric artery) in patients with Siewert type II AEG compared to stations 1, 2 (left paracardia), 3, 7, and 9 (along the celiac artery) in those with Siewert type III AEG.

**Table 3:** Frequency of lymph node metastasis and 5-year survival in patients with Siewert type II adenocarcinoma of the esophagogastric junction based on lymph node station

Lymph node station	Number of patients with metastatic nodes	Number of patients in whom the station was dissected	Incidence of lymph node metastasis (%)	5-year survival rate of patients with metastatic nodes (%)	IEBLD
1	36	92	39.1	36.6	14.3
2	12	92	13.0	15.9	2.1
3	34	92	37.0	45.4	16.8
4sa	2	92	2.2	0	0
4sb	2	92	2.2	50.0	1.1
4d	0	73	0	0	0
5	2	73	2.7	0	0
6	0	73	0	0	0
7	20	92	21.7	40.6	8.8
8a	3	85	3.5	50.0	1.8
9	15	87	17.2	22.9	3.9
10	3	52	5.8	0	0
11p	10	76	13.2	19.0	2.5
11d	2	65	3.1	0	0
12a	0	14	0	0	0
19	4	23	17.4	0	0
20	2	21	9.5	50.0	4.8

*IEBLD* Index of estimated benefit from lymph node dissection

**Table 4:** Frequency of lymph node metastasis and 5-year survival in patients with Siewert type III adenocarcinoma of the esophagogastric junction based on lymph node station

Lymph node station	Number of patients with metastatic nodes	Number of patients in whom the station was dissected	Incidence of lymph node metastasis (%)	5-year survival rate of patients with metastatic nodes (%)	IEBLD
1	21	40	52.5	52.2	27.4
2	8	40	20.0	50.0	10.0
3	20	40	50.0	43.8	21.9
4sa	1	40	2.5	0	0
4sb	3	40	7.5	50.0	3.8
4d	4	38	10.5	25.0	2.6
5	2	38	5.3	50.0	2.6
6	0	38	0	0	0
7	8	40	20.0	40.0	8.0
8a	2	38	5.3	0	0
9	6	39	15.4	33.3	5.1
10	3	31	9.7	0	0
11p	4	36	11.1	0	0
11d	2	30	6.7	0	0
12a	0	12	0	0	0
19	1	5	20	0	0
20	0	2	0	0	0

*IEBLD* Index of estimated benefit from lymph node dissection

On the contrary, the IEBLDs of stations located far from the EGJ were low. It was zero in stations 4d, 5, 6, and 12a (along the proper hepatic artery) in patients with Siewert type II AEG compared to only stations 6 and 12a in patients with Siewert type III AEG. The IEBLD of station 10 (splenic hilum) was zero in both groups.

In this study, we did not calculate IEBLDs of paraaortic and mediastinal lymph nodes, because only a few patients underwent lymph node dissection of these stations (paraaortic lymph node; 9 patients, mediastinal lymph node; 17 patients).

## Discussion

In this series of 132 patients with AEG, 69.7 % were classified as Siewert type II and 30.3 % as Siewert type III. The incidence of lymph node metastasis was high in both groups, and the IEBLDs of stations located near the EGJ were similar between the groups, while those located far from the EGJ were different between the groups. Our findings therefore indicate that the optimal strategy for lymph node dissection could differ between Siewert type II and III AEGs.

In the present study, the incidence of lymph node metastasis was 42.9 % in pT1 AEG and 74.0 % in pT2–4 AEG, which is higher than previously reported incidence ranges of 11.3–15.1 and 48.1–66.5 % in early and advanced gastric cancer, respectively [16–19]. We therefore considered that complete retrieval of susceptible stations for metastasis was necessary to improve survival outcomes in patients with AEG and sought to establish the optimal intra-abdominal lymphadenectomy strategies for Siewert type II and III AEGs.

In the present study, tumors infiltrated deeper and the pathological stage was more advanced in Siewert type III AEG than in Siewert type II AEG, as reported in previous studies [6]. Theoretically, the epicenter of Siewert type III AEG is far from the EGJ compared to that of Siewert type II AEG; thus, Siewert type III AEG must be larger in diameter to infiltrate the junction, as was the case in the present study, resulting in the deeper tumor infiltration and advanced stage observed. However, the survival outcome was not different between Siewert type II and III AEGs despite of the difference in stage distributions. The technical difficulty of surgery for Siewert type II AEG, including mediastinal lymph node dissection, compared to Siewert type III AEG may be a possible reason for this paradoxical result. In addition, because the incidence of Siewert type II AEG had been low in Japan, the appropriate treatment strategy for the disease might not be established particularly in the early period of the present study, resulting in inadequate mediastinal lymph node dissection and worse survival outcomes.

In the present study, the IEBLDs of stations 1, 3, and 7 were over 5.0 in Siewert type II AEG, consistent with previous studies showing high IEBLDs in paracardial and lesser curvature lymph nodes [8, 9, 11]. However, the IEBLDs of Siewert type III AEGs have not been investigated in detail before [6, 20, 21], and to the best of our knowledge, this is the first study to fully investigate IEBLDs of Siewert type III, although Kodera et al. [13] investigated the incidence of lymph node metastasis and survival outcomes in patients with positive nodes without calculating IEBLDs. The present study also found high IEBLDs in paracardial and lesser curvature lymph nodes, indicating that dissection of these nodes is inevitable in both Siewert type III and II AEGs.

In Siewert type II AEG, the IEBLDs of the lower perigastric lymph nodes (station 4d, 5, and 6) were zero in the present study. Yamashita et al. [8] also reported the low therapeutic value of lower perigastric lymph node dissection for Siewert type II AEG. Such a dissection, omitting the lower perigastric lymph nodes, might preserve the distal part of the stomach, although whether proximal gastrectomy really provides some benefits over total gastrectomy, such as a better postoperative quality of life, remains to be clarified [9]. Further comparative study of proximal gastrectomy and total gastrectomy is necessary to resolve this issue.

On the other hand, the present study showed that IEBLDs of the lower perigastric lymph nodes were not zero in Siewert type III AEG, and a past study demonstrated lymphatic flow from the middle third of the stomach to lower perigastric lymph nodes [22]. It is therefore possible that the lower perigastric lymph nodes could be involved in cases with tumor infiltrated to the middle third of the stomach even if the primary tumor epicenter is located within the upper third of the stomach. Indeed, in Siewert type III cases, the tumors were large enough to infiltrate to the middle third of the stomach. Therefore, we consider the IEBLDs of station 4d and 5 were not zero, and total gastrectomy is necessary for Siewert type III AEG. Consistent with this, a previous report also recommended total gastrectomy with distal esophagectomy including lower perigastric lymph node dissection for Siewert type III AEG [23].

We previously reported that the IEBLD of the splenic hilar lymph nodes was zero in patients with Siewert type II AEG who underwent total gastrectomy with D2 lymph node dissection [11]. The present study thus mirrored the previous result in patients with Siewert type II AEG. Additionally, the IEBLD of the splenic hilar lymph nodes was zero in patients with Siewert type III AEG in the present study. Reported IEBLDs of the splenic hilar lymph nodes range from 0.7 to 2.2, and most authors considered splenic hilar lymph node dissection can be omitted without decreasing curability [6, 8, 9]. In Japan, a large-scale randomized control trial (JCOG0110) to evaluate splenectomy for proximal gastric cancer without involvement of the greater curvature is currently in progress, and we should await the results to clarify this aspect of AEG management [24].

The present retrospective study has limitations. First, the value of mediastinal lymph node dissection was not evaluated because we did not perform such a procedure routinely, particularly in the early study period. Although the latest JGCA guidelines recommend lower mediastinal lymph node dissection for patients with advanced gastric cancer invading the esophagus [14], the former JGCA guidelines used during the early study period did not mention this issue. We currently perform lower mediastinal lymph node dissection for advanced AEG and should evaluate the value of lower mediastinal lymph node dissection in the near future. Second, we did not evaluate the value of para-aortic lymph node dissection. In a randomized controlled trial investigating the value of left thoracotomy for AEG (JCOG9502), the incidence of para-aortic lymph node metastasis (12.0 %) was as high as for other nodes [25]. In addition, Mine et al. [10] reported that the rate of lymph node metastasis of para-aortic lymph nodes around the left renal vein (17.0 %) was similar to that of some suprapancreatic lymph nodes (12.7–16.5 %) in Siewert type II AEGs. In both reports, the IEBLD of the para-aortic lymph nodes was high. Therefore, the value of para-aortic lymph node dissection for AEG should be clarified in the future [10, 25].

In conclusion, IEBLDs for each lymph node station were similar between Siewert type II and III AEGs except for the lower perigastric lymph nodes. According to the results of the present study, total gastrectomy should be selected as a standard treatment for Siewert type III AEG. In contrast, preservation of the distal part of the stomach may be an acceptable procedure in patients with Siewert type II AEG, because the present study did not show survival benefit for lower perigastric lymph node dissection.
